# Comparative Effectiveness and Safety of Direct Oral Anticoagulants vs Warfarin Among Obese Patients With Atrial Fibrillation

**DOI:** 10.1016/j.cjco.2022.01.002

**Published:** 2022-01-13

**Authors:** Laurie-Anne Boivin-Proulx, Brian J. Potter, Marc Dorais, Sylvie Perreault

**Affiliations:** a Centre de Recherche du Centre Hospitalier de l’Université de Montréal (CRCHUM), Montreal, Quebec, Canada; b Centre Cardiovasculaire du Centre Hospitalier de l’Université de Montréal (CHUM), Montreal, Quebec, Canada; c StatSciences Inc., Notre-Dame-de-l'Île-Perrot, Quebec, Canada; d Faculty of Pharmacy, Université de Montréal, Montreal, Quebec, Canada; e Chaire Sanofi sur l'utilisation des médicaments, Université de Montréal, Montreal, Quebec, Canada; f Centre de recherche en santé publique (CReSP), partenaire CIUSSS du Centre-Sud-de-l’Île-de-Montréal et l’Université de Montréal, Montreal, Quebec, Canada

## Abstract

**Background:**

Obese patients are underrepresented in clinical trials assessing the comparative effectiveness and safety of use of direct oral anticoagulants vs use in atrial fibrillation (AF) patients.

**Methods:**

Using data from Quebec provincial administrative databases, for the years2010-2017, we created a retrospective cohort of patients with inpatient or outpatient coding for AF and obesity who were newly prescribed an oral anticoagulant. The primary safety outcome was a composite of intracranial, gastrointestinal, and major bleeding from other sites, and the primary effectiveness outcome was a composite of ischemic stroke, systemic embolism, acute myocardial infarction, and death in the first year after oral anticoagulant initiation. Treatment groups were compared using inverse-probability-of-treatment-weighting Cox proportional-hazards models.

**Results:**

A total of 2263 patients were included, of whom 1253, 403, and 539 filled a warfarin, standard-dose rivaroxaban, and standard-dose apixaban prescription, respectively. Standard-dose rivaroxaban was associated with a similar composite safety (hazard ratio [HR] 0.91; 95% confidence interval [CI] 0.44-1.91) and composite effectiveness risk (HR 1.42; 95% CI 0.99-2.04) compared to warfarin, whereas standard-dose apixaban was associated with a lower composite safety (HR 0.40; 95% CI 0.16-0.98) and similar composite effectiveness risk (HR 0.96; 95% CI 0.67-1.39).

**Conclusion:**

Use of direct oral anticoagulants in obese AF patients was associated with a similar effectiveness and safety profile to that of warfarin use.

## Résumé

**Introduction:**

Les patients obèses sont sous-représentés dans les essais cliniques qui portent sur l’évaluation de l’efficacité comparative et l’innocuité de l’utilisation d’anticoagulants oraux directs *vs* leur utilisation chez les patients atteints de fibrillation auriculaire (FA).

**Méthodes:**

À l’aide des données de la base de données administratives provinciales du Québec, des années 2010-2017, nous avons créé une cohorte rétrospective de patients dont le codage des séjours hospitaliers et en ambulatoire étaient la FA et l’obésité qui avaient récemment reçu une ordonnance d’anticoagulants oraux. Le principal critère d’évaluation de l’innocuité était un critère composite qui associait les hémorragies intracrâniennes, gastro-intestinales et majeures d’autres sites, et le principal critère d’évaluation de l’efficacité était un critère composite d’accident vasculaire cérébral, d’embolie systémique, d’infarctus aigu du myocarde et de décès dans la première année après l’amorce des anticoagulants oraux. Nous avons comparé les groupes de traitement à l’aide des modèles à risques proportionnels de Cox basés sur la probabilité inverse de pondération de traitement.

**Résultats:**

Nous avons retenu un total de 2 263 patients, dont 1 253, 403 et 539 ont pris de façon respective les médicaments prescrits suivants : la warfarine, le rivaroxaban à la posologie standard et l’apixaban à la posologie standard. Le rivaroxaban à la posologie standard était associé à un risque de survenue d’un événement compris dans le critère composite de l’innocuité (ratio d’incidence approché [RIA] 0,91; intervalle de confiance [IC] à 95 % 0,44-1,91) et un risque de survenue d’un événement compris dans le critère composite de l’efficacité (RIA 1,42; IC à 95 % 0,99-2,04) similaires par rapport à la warfarine, tandis que l’apixaban à la posologie standard était associé à un risque de survenue d’un événement compris dans le critère composite de l’innocuité plus faible (RIA 0,40; IC à 95 % 0,16-0,98) et un risque de survenue d’un événement compris dans le critère composite de l’efficacité similaire (RIA 0,96; IC à 95 % 0,67-1,39).

**Conclusion:**

L’utilisation d’anticoagulants oraux directs chez les patients obèses atteints de FA était associée à un profil d’efficacité et d’innocuité similaire à celui de l’utilisation de la warfarine.

Obesity is an independent risk factor for atrial fibrillation (AF), with a 10%-29% excess risk of incident AF for every 5-unit increase in body mass index (BMI).^1^ However, somewhat paradoxically, BMI is independently associated with a lower risk of stroke/systemic embolism (SE), and a higher bleeding risk.^2^^,^^3^ Because of these risk factors, the risk-benefit threshold for oral anticoagulant (OAC) prescription may not occur at the same point in obese and nonobese patients. This clinical conundrum is further compounded by both a lack of robust pharmacokinetic data to guide the use of direct oral anticoagulants (DOACs) in patients with extreme BMIs and a paucity of clinical data to support DOAC prescription in obese populations.^4^

In 2016, an obesity subgroup analysis of phase 3 clinical trial data on DOACs was conducted by the Scientific and Standardization Committee of the International Society of Thrombosis and Haemostasis to look at the efficacy and safety of DOAC use in obese patients.^4^ The committee concluded that DOACs are safe and effective, but only up to a BMI of ≤ 40 kg/m^2^ or a body weight ≤ 120 kg.^4^ Citing very limited data regarding DOAC use in severe obesity (BMI > 40 kg/m^2^ or > 120 kg), and concerns for possible decreased peak concentrations and shorter drug half-lives, the committee recommended that DOACs not be used in severely obese patients, unless specific monitoring of DOAC activity is ensured.^4^

Subsequently, a number of analyses of landmark DOAC AF trials have evaluated the impact of BMI on drug efficacy and safety, but most did not include a significant proportion of severely obese patients.^2^^,^^3^^,^^5^, ^6^, ^7^, ^8^ Given the pharmacokinetic issues regarding DOAC use in severely obese patients, and the limitations of available clinical trial data, large-scale real-world studies addressing the comparative effectiveness and safety of different DOAC regimens among obese AF patients are required. We therefore conducted a retrospective analysis of the safety and effectiveness of use of a DOAC vs use of warfarin in obese AF patients, using province-wide Quebec healthcare claims databases.

## Methods

### Data sources

Provincial administrative databases of hospital discharges (Med-Echo) and public medical services administered by the Régie de l’Assurance Maladie du Québec (RAMQ) were linked using encrypted health insurance numbers and used to derive the study cohort.^9^, ^10^, ^11^, ^12^ Ethics approval of the project was obtained from the University of Montreal Ethics Committee.

### Population

The RAMQ and Med-Echo databases were queried to identify adult patients aged ≥ 18 years with inpatient or outpatient diagnostic coding of AF from January, 2010 to December, 2017, using International Classification of Diseases, 9th edition (ICD-9) codes (427.3, 427.31, or 427.32) or the ICD 10th edition (ICD-10) code (I48).^13^^,^^14^ In case of more than one eligible AF admission, the date of the first AF diagnosis was used as the eligible date. ICD-9 diagnostic codes for AF have a median positive predictive value (PPV) of 89%.^15^ Patients with ≥ 1 diagnosis of obesity based on ICD-9 codes (278.00, 278.0, or V77.8) or ICD-10 codes (E66.9, E66.01, or Z13.89) were subsequently identified.^16^ ICD-9 and ICD-10 codes for obesity have a PPV of 92%.^17^

Among identified obese AF patients, those who initiated OAC treatment within 1 year from the AF diagnosis were retained. The patients needed to be new users, defined as having not been exposed to any OAC in the year before the index claim date. The date of the index OAC claim following AF diagnosis was defined as the date of cohort entry. Patients were required to have been enrolled in the provincial drug insurance plan for a minimum of 12 months prior to the index claim. Patients who resided in long-term care facilities that typically provide medications to patients, and those not covered by the Quebec drug insurance plan, were therefore excluded. We also excluded patients with venous thromboembolism within 1 year of cohort entry, end-stage chronic kidney disease, dialysis for more than 3 months, kidney transplant or coagulation deficiency within 3 years of cohort entry, or cardiac valvular replacement within 5 years of cohort entry. Additionally, patients were excluded if they had undergone recent procedures that might influence OAC treatment, including angioplasty, coronary bypass surgery, cerebrovascular and valvular procedures in the 3 months prior to cohort entry, and hip, pelvic, or knee fracture in the 6 weeks prior to cohort entry.

### Exposures

Patient treatment with OACs was verified using fill dates and days supplied for each claim. We define 2 types of exposure—intent-to-treat (ITT; primary analysis) and undertreatment (UT). In the ITT analysis, patients were assumed to persist with their first prescribed OAC for 365 days (end of the study period).

The exposure of UT was defined as continuing treatment as long as they filled prescriptions within 30 days of the end of the last treatment episode. So, patients were censored at the time of discontinuation of treatment, or of switching to another OAC or another dosage. Allowing a gap in treatment of up to 30 days is a reasonable metric because of the short half-life of DOACs. Consequently, we chose 30 days as the allowable gap, corresponding to an adherence of 92% or more over the fixed 12-month exposure assessment period.

With either exposure definition, patients were censored at the time of enrollment in a nongovernmental drug coverage plan, admission to a long-term care facility, hospitalization for > 15 days, or if they experienced a safety or efficacy outcome, whichever came first. Patients’ OAC exposure and censoring status were updated at 30-day intervals.

### Outcome measures

The primary safety outcome was a composite of major bleeding events defined by intracranial hemorrhage (ICH), gastrointestinal bleeding, and major bleeding from other sites. The primary effectiveness outcome was defined as a composite of ischemic stroke, SE, acute myocardial infarction (AMI), and all-cause mortality. The individual components of the safety and effectiveness outcome were evaluated in a secondary analysis. Transient ischemic attack was excluded from the main effectiveness composite outcome because of the inherent difficulties in retrospectively validating it as a diagnosis. Furthermore, we defined an irreversible events composite outcome as ischemic stroke, hemorrhagic stroke, ICH, AMI, and all cause-mortality. ICD-9 and ICD-10 codes for the primary diagnosis of inpatient claims were used to identify outcomes (Supplemental Table S1) and have been shown to have good validity, with PPV > 80%.^18^^,^^19^

### Patient demographics and clinical characteristics

Demographic data were documented at cohort entry. Comorbidities were determined using inpatients’ and outpatients’ ICD-9 and ICD-10 diagnoses occurring in the 3 years preceding the index date.^14^^,^^18^^,^^20^ Patients’ demographic characteristics and comorbidities were used to evaluate the CHADS_2_ (**C**ongestive Heart Failure, **H**ypertension, **A**ge ≥ 75, **D**iabetes, and Prior **S**troke/Transient Ischemic Attack) score (Supplemental Tables S2 and S3), the CHA_2_DS_2_-VASc (**C**ongestive Heart Failure, **H**ypertension, **A**ge [≥ 75 Years], **D**iabetes Mellitus, **S**troke, **V**ascular Disease, **A**ge [65-74] Years, **S**ex **C**ategory [Female]) score (Supplemental Tables S2 and S4) and the modified HAS-BLED (**H**ypertension, **A**bnormal Renal/Liver Function, **S**troke, **B**leeding History or Predisposition, **L**abile INR, **E**lderly [> 65 Years], **D**rugs/Alcohol Concomitantly) score (Supplemental Tables S2 and S5). The Charlson Comorbidity Index was also calculated for each patient.^21^

### Statistical analyses

Descriptive statistics were used to summarize characteristics of the patients according to the type of OAC they initially received. Thereafter, we adopted an inverse-probability-of-treatment-weighting (IPTW) method to account for differences in patient characteristics between treatment groups. A multivariate logistic regression model was used to estimate the probability of being in the observed treatment group, conditional on all baselines covariates. Adjusted descriptive statistics were also used to summarize baseline characteristics of each IPTW cohort. Absolute standardized differences of baseline characteristics between unadjusted and IPTW cohorts > 10% were considered meaningful.

Dose-specific DOAC groups were compared to warfarin in the IPTW cohort. Outcome cumulative incidence per 100 person-years is reported. Hazard ratios (HRs) were estimated using Cox proportional hazards models for the primary safety and effectiveness composites, as well as the irreversible outcomes composite. Ultimately, however, Cox regression was applied to only the comparisons between rivaroxaban or apixaban and warfarin, because of low sample-size numbers in other DOAC treatment groups. HRs are reported with their corresponding 95% confidence interval (CI).

All analyses were performed using SAS 9.4 statistical software (SAS Institute, Cary, NC).

## Results

### Demographics and clinical characteristics

**Figure 1:**
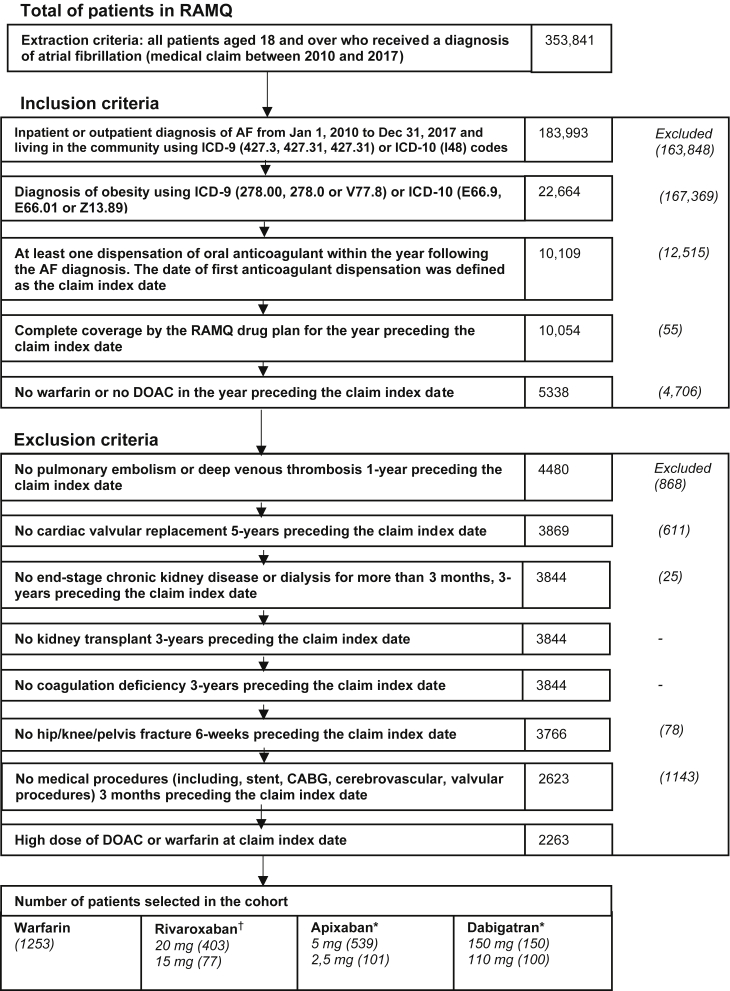
Flowchart of study design, and patients in the study cohort. Given insufficient sample size, inverse-probability-of-treatment weighting and assessment of estimated hazard ratios for outcomes using Cox proportional hazard models were restricted to warfarin vs standard-dose rivarobaxan (20 mg once daily) and warfarin vs apixaban (5 mg by mouth once daily). AF, atrial fibrillation; CABG, coronary artery bypass grafting; Dec, December; DOAC, direct oral anticoagulant; ICD-9, International Classification of Diseases, 9th edition; ICD-10, ICD, 10th edition; Jan, January; OAC, oral anticoagulant; RAMQ, Régie d’Assurance Maladie du Québec (Quebec administrative databases). ∗Twice daily. ^†^Once daily.

A flowchart of the study design and the patients in the study cohort is shown in Figure 1. A total of 2623 patients were included, among whom 1253, 403, and 539 patients filled a new warfarin, rivaroxaban (20 mg die), and apixaban (5 mg twice daily) prescription, respectively, between 2010 and 2017. Unadjusted and adjusted patient characteristics are in shown in Supplemental Table S6 and Supplemental Tables S7-S8, respectively. Before adjustment, standard-dose rivaroxaban users were younger (69.30 ± 8.76 vs 74.54 ± 9.80 years), and had a lower Charlson score (4.13 ± 3.26 vs 5.21± 3.25), CHADS_2_ score (2.10 ± 1.22 vs 2.78 ± 1.26), and HAS-BLED score (2.81 ± 1.39 vs 3.54 ± 1.40), compared to warfarin users. Standard-dose apixaban users were also younger (73.50 ± 8.63 vs 74.54 ± 9.80 years), and had a lower Charlson score (4.47 ± 3.34 vs 5.21 ± 3.25), CHADS_2_ score (2.48 ± 1.20 vs 2.78 ± 1.26), and HAS-BLED score (3.10 ± 1.36 vs 3.54 ± 1.40), compared with warfarin users.

### Cumulative incidence rates within IPTW cohort

**Figure 2:**
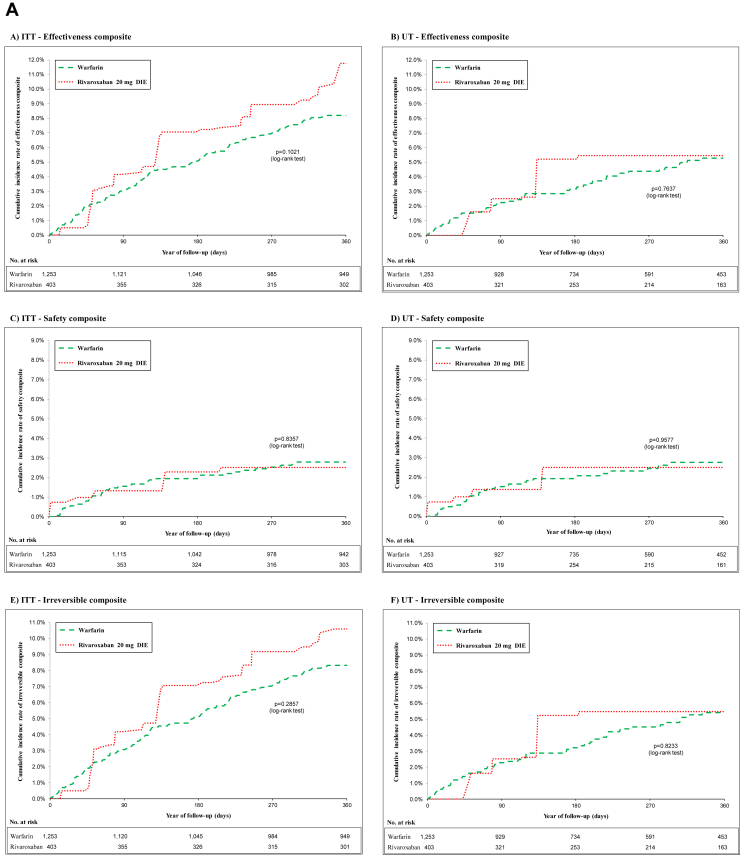

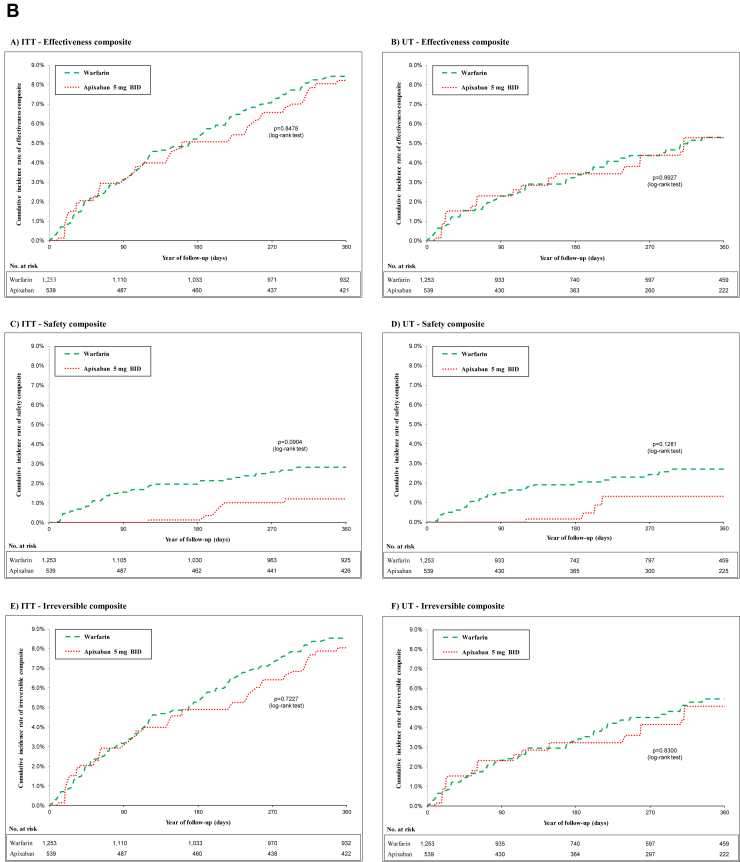
(**A**) Cohort of warfarin vs standard-dose rivaroxaban using inverse-probability-of-treatment weighting at intent-to-treat (ITT) and undertreatment (UT) analysis: primary safety, secondary effectiveness, and irreversible outcomes. (**B**) Cohort of warfarin vs standard-dose apixaban using inverse-probability-of-treatment weighting at ITT and UT analysis primary safety, secondary effectiveness, and irreversible outcomes. BID, twice daily; DIE, once daily.

As shown in Table 1, patient characteristics were well balanced between IPTW treatment groups. Figure 2, A and B shows the cumulative incidence curves for the composite outcomes of standard-dose rivaroxaban and apixaban, compared with warfarin, in both the ITT and UT analyses. Yearly rates for safety, effectiveness, and irreversible events composites for standard-dose rivaroxaban and apixaban vs warfarin are presented in Table 2.

**Table 1:** Cohort using inverse-probability-of-treatment weighting for warfarin vs standard-dose rivaroxaban and apixaban

	Warfarin vs rivaroxaban cohort			Warfarin vs apixaban cohort		
Characteristic	Warfarin (n = 1253)	Rivaroxaban 20 mg once daily (n = 403)	Absolute standardized difference	Warfarin (n = 1253)	Apixaban 5 mg twice daily (n = 539)	Absolute standardized difference
Age, y ^‡^	72.83 ± 11.07	71.91 ± 8.09	0.11	74.25 ± 9.87	74.22 ± 8.26	0.00
Male	551 (43.71)	178 (45.57)	0.04	547 (43.68)	239 (44.45)	0.02
CHADS_2_ score	2.60 ± 1.28	2.60 ± 1.28	0.00	2.69 ± 1.25	2.71 ± 1.22	0.01
HAS-BLED score	3.35 ± 1.44	3.35 ± 1.43	0.00	3.42 ± 1.38	3.50 ± 1.48	0.05
Charlson score	4.92 ± 3.30	5.11 ± 3.35	0.06	5.00 ± 3.30	5.15 ± 3.49	0.04
**Comorbidities including index hospitalization and 3-y prior index**						
Hypertension	1100 (87.28)	344 (88.06)	0.02	1119 (89.26)	485 (90.22)	0.03
Coronary artery disease	710 (56.35)	217 (55.48)	0.02	710 (56.63)	304 (56.51)	0.00
AMI	157 (12.45)	45 (11.51)	0.03	158 (12.59)	85 (15.75)	0.09
Chronic heart failure	544 (43.12)	174 (33.52)	0.03	561 (44.69)	235 (43.71)	0.02
Valvular heart disease	237 (18.82)	75 (19.30)	0.01	234 (18.62)	97 (18.10)	0.01
Cardiomyopathy	93 (7.39)	26 (6.65)	0.03	94 (7.47)	36 (6.76)	0.03
Other cardiac dysrhythmias	225 (17.83)	75 (19.20)	0.04	229 (18.27)	113 (21.01)	0.07
Peripheral arterial disease	263 (20.85)	88 (22.57)	0.04	250 (29.96)	207 (20.00)	0.00
Dyslipidemia	772 (61.20)	243 (62.09)	0.02	766 (61.06)	333 (61.99)	0.02
Diabetes	756 (59.93))	237 (60.72)	0.02	766 (61.09)	334 (62.26)	0.02
Major bleeding	372 (29.50)	112 (28.54)	0.02	378 (30.17)	174 (32.36)	0.05
Chronic renal failure	540 (42.85)	163 (41.72)	0.02	555 (44.28)	244 (45.40)	0.02
Acute renal failure	5375 (29.76)	111 (28.40)	0.03	387 (30.84)	173 (32.38)	0.03
Liver disease	32 (2.55)	12 (2.97)	0.03	34 92.74)	15 (2.73)	0.00
Chronic obstructive pulmonary disease	587 (46.58))	194 (49.63)	0.06	568 (45.31)	254 (47.24)	0.04
Systemic embolism	32 (2.51)	11 (2.81)	0.02	33 (2.62)	15 (2.86)	0.01
*Helicobacter pylori* infection	11 (0.85)	5 (1.18)	0.03	12 (0.92)	6 (1.13)	0.02
Depression	150 (11.87)	49 (12.54)	0.02	141 (11.24)	71 (13.21)	0.06
Hypothyroidism	281 (22.31)	78 (20.05)	0.06	276 (22.01)	130 (24.24)	0.05
Neurologic disorder	243 (19.27)	70 (17.88)	0.04	244 (19.46)	100 (18.60)	0.02
Malign cancer	244 (19.34)	82 (21.10)	0.04	247 (19.67)	107 (19.86)	0.00
**Medical procedures (3 y prior to entry)**						
Cardiac catheterization	70.16 (5.57)	25 (6.37)	0.03	72 (5.75)	36 (6.61)	0.04
PCI—stent	45 (3.59)	15 (3.80)	0.01	42 (3.32)	19 (3.56)	0.01
CABG	18 (1.45)	5 (1.39)	0.01	23 (1.83)	10 (1.87)	0.00
Implantable cardiac devices	9 (0.72)	0 (0.00)	0.12	8.39 (0.67)	0 (0.00)	0.11
**Medications (Initiation or 2 wk prior to entry)**						
Statin	675 (53.52))	224 (57.31)	0.08	662 (52.76)	291 (54.25)	0.03
Antiplatelet (excluding low-dose ASA)	75 (5.94)	28 (7.14)	0.05	68 (5.45)	28 (5.26)	0.01
Low-dose ASA	441 (34.97)	146 (37.48)	0.05	417 (33.24)	178 (33.09)	0.00
Proton pump inhibitors	536 (42.50)	159 (40.78)	0.03	537 (42.78)	233 (43.43)	0.01
NSAIDs	30 (2.40)	17 (4.30)	0.11	26 (2.07)	10 (1.90)	0.01
Digoxin	157 (12.44)	47 (11.96)	0.01	158 (12.57)	59 (10.95)	0.05
Amiodarone	122 (9.64)	34 (8.64)	0.04	126 (10.08)	58 (10.87)	0.03
Antidepressants	110 (8.76)	34 (8.62)	0.01	112 (8.94)	57 (10.61)	0.06
B-blockers	760 (60.30))	236 (60.42)	0.00	755 (60.21)	320 (59.62)	0.01
Calcium-channel blockers	525 (41.67)	164 (42.06)	0.01	507 (40.43)	224 (41.67)	0.03
Inhibitors of renin-angiotensin system	540 (42.80)	181 (46.22)	0.07	548 (43.70)	231 (42.98)	0.01
Loop diuretics	526 (41.69)	166 (42.49)	0.02	543 (43.32)	225 (41.95)	0.03
Diuretics	621 (49.30)	194 (49.68)	0.01	637 (50.82)	271 (50.42)	0.01
Antidiabetics	483 (38.31)	149 (39.09)	0.00	492 (39.21)	227 (42.32)	0.06
**Medical service (1 y prior to entry)**						
Number of specialty visits	1.11 ± 2.05	1.03 ± 2.59	0.03	1.16 ± 2.05	1.22 ± 2.08	0.03
Number of family physician visits	1.30 ± 2.78	1.34 ± 3.61	0.01	1.22 ± 2.71	1.52 ± 4.05	0.09
**Hospital service (3 y prior to entry)**						
Number of emergency visits	3.22 ± 2.73	3.13 ± 2.50	0.04	3.28 ± 2.79	3.32 ± 3.29	0.01
Number of all-cause hospital admission	2.35 ± 1.91	2.44 ± 1.79	0.05	2.34 ± 1.90	2.49 ± 2.11	0.05

Values are mean ± standard deviation, or n (%), unless otherwise indicated.

AMI, acute myocardial infarction; ASA, acetylsalicylic acid; CABG, coronary artery bypass grafting; CHADS_2_, **C**ongestive Heart Failure, **H**ypertension, **A**ge ≥ 75, **D**iabetes, and Prior **S**troke/TIA_2_; HAS-BLED, **H**ypertension, **A**bnormal Renal/Liver Function, **S**troke, **B**leeding History or Predisposition, **L**abile INR, **E**lderly (> 65 Years), **D**rugs/Alcohol Concomitantly; NSAID, nonsteroidal anti-inflammatory drug; PCI, percutaneous coronary intervention.

**Table 2:** Rate of clinical events of warfarin use vs DOAC use during 1-year period of follow-up using inverse-probability-of-treatment weighting

Endpoint	Analysis	DOAC	Warfarin	HR (95% CI)	*P*
**Rivaroxaban 20 mg once daily vs warfarin**					
Safety composite	ITT	2.7	3.0	0.91 (0.44–1.91)	0.81
	UT	3.2	3.3	0.98 (0.45–2.13)	0.95
Effectiveness composite	ITT	12.6	8.9	1.42 (0.99–2.04)	0.06
	UT	6.6	6.1	1.10 (0.63–1.90)	0.74
Irreversible outcomes	ITT	11.5	9.0	1.27 (0.88–1.85)	0.21
	UT	6.6	6.2	1.07 (0.62–1.86)	0.80
**Apixaban 5.0 mg once daily vs warfarin**					
Safety composite	ITT	1.2	3.0	0.40 (0.16–0.98)	0.05
	UT	1.3	3.3	0.40 (0.15–1.07)	0.07
Effectiveness composite	ITT	8.7	9.1	0.96 (0.67–1.39)	0.82
	UT	6.0	6.1	1.00 (0.60–1.66)	0.99
Irreversible outcomes	ITT	8.5	9.3	0.93 (0.64–1.34)	0.68
	UT	5.8	6.3	0.94 (0.56–1.57)	0.81

CI, confidence interval; DOAC, direct oral anticoagulant; HR, hazard ratio; ITT, intention to treat; UT, under treatment.

### Adjusted hazard of composite outcomes with rivaroxaban

As shown in Figure 2A, using an ITT analysis with warfarin as the reference group, standard-dose rivaroxaban was associated with a similar composite safety risk (HR 1.94; 95% CI 0.82-4.58), but a trend toward poorer effectiveness (HR 1.94; 95% CI 0.99-2.04). The latter was driven by an increased risk of SE (HR 8.20; 95% CI 1.47-45.69) and AMI (HR 2.19; 95% CI 2.19-4.30) with standard-dose rivaroxaban. No significant difference was observed in the risk of composite irreversible events (HR 1.25; 95% CI 0.88-1.85).

In the UT analysis, standard-dose rivaroxaban was associated with a similar composite safety risk (HR 0.98; 95% CI 0.45-2.13). Standard-dose rivaroxaban had a similar composite effectiveness risk (HR 1.10; 95% CI 0.63-1.90) and composite irreversible events risk (HR 0.63; 95% CI 0.63-1.90), compared with warfarin. Analyses of the individual components of the composite outcome are presented in Supplemental Table S9.

### Adjusted hazard of composite outcomes with apixaban

As shown in Figure 2B, in an ITT analysis with warfarin as the reference group, standard-dose apixaban was associated with a lower composite safety risk (HR 0.40; 95% CI 0.16-0.98). No significant difference was seen in the risk of composite effectiveness (HR 0.96; 95% CO 0.67-1.39) or of composite irreversible events (HR 0.93; 95% CI 0.64-1.34).

In UT analyses, standard-dose apixaban use was again associated with a trend toward reduction in composite safety risk, compared with warfarin use (HR 0.40; 95% CI 0.15-1.07), with a similar effectiveness profile (composite effectiveness HR 0.94; 95% CI 0.56-1.57; composite irreversible events HR 1.00; 95% CI 0.60-1.57). Analyses of the individual components of the composite outcome are presented in Supplemental Table S10.

## Discussion

This analysis is the largest Canadian real-world study specifically addressing the comparative safety and effectiveness of various DOAC regimens among obese AF patients. The principal finding is that DOACs are indeed of comparable effectiveness to warfarin in obese patients, and they offer similar or better safety profiles.

Current AF guidelines recommend DOACs over warfarin when OAC therapy is indicated, in most patients with nonvalvular AF (NVAF),^22^, ^23^, ^24^ based on the results of several large randomized controlled trials (RCTs) demonstrating that DOACs are non-inferior or superior in reducing the risk of AF-associated stroke or SE, with a lower or similar major bleeding risk compared with that of warfarin (in addition to the logistic advantages of DOACs, compared with dose-adjusted warfarin).^6^^,^^25^, ^26^, ^27^ However, current AF guidelines provide little guidance on DOAC usage in obese populations.

A post hoc analysis of the **R**ivaroxaban **O**nce Daily Oral Direct Factor Xa Inhibition **C**ompared With Vitamin **K** Antagonist for Prevention of Stroke and **E**mbolism **T**rial in **A**trial **F**ibrillation (ROCKET-AF) trial, including 620 severely obese patients (BMI ≥ 40 kg/m^2^ or > 120 kg), found comparable safety and effectiveness of rivaroxaban vs warfarin across various BMI subgroups, with no interaction between BMI subgroups (*P* = 0.69 and *P* = 0.31, respectively).^3^^,^^27^ The post hoc analysis of the **E**ffective A**n**ticoa**g**ulation With Factor X**a** Next **Ge**neration in **A**trial **F**ibrillation-**T**hrombolysis **i**n **M**yocardial **I**nfarction 48 (ENGAGE AF-TIMI 48), including 1149 patients with a BMI ≥ 40 kg/m^2^, also found comparable safety and effectiveness of edoxaban vs warfarin across various BMI subgroups, with no interaction between BMI subgroups (*P* = 0.16 and *P* = 0.81, respectively).^28^ A post hoc analysis of the **A**pixaban for **R**eduction **i**n **St**roke and **O**ther **T**hromboembo**l**ic **E**vents in Atrial Fibrillation (ARISTOTLE) study, including 920 severely obese patients (> 120 kg) found that the superiority of apixaban vs warfarin was consistent across the weight spectrum for stroke/SE (*P* = 0.64), but that the major bleeding risk reduction was greater in the low-range (< 60 kg) and mid-range (60-120 kg) weights (*P* = 0.02).^8^

Although the Scientific and Standardization Committee of the International Society of Thrombosis and Haemostasis published guidance on DOAC use in obese patients based on a subgroup analysis of phase 3 DOAC clinical trial data,^4^ the committee did not provide guidance for patients with a BMI > 40 kg/m^2^ or a weight > 120 kg, instead stating that DOACs should not be used in severely obese patients unless specific monitoring of DOAC activity can be ensured.^4^

Since then, some observational studies have provided evidence supporting the use of DOACs in obese patients.^29^, ^30^, ^31^, ^32^, ^33^, ^34^ Costa et al. analyzed electronic health record data of obese NVAF patients, including 18,034 who were severely obese ( > 120 kg), and they observed that rivaroxaban-treated patients (*n* = 38,848) had a 17% lower risk of stroke/SE (HR 0.83; 95% CI 0.73-0.94), and 18% had a lower risk of major bleeding (HR 0.82; 95% CI 0.75-0.89), compared with warfarin-treated patients (*n* = 57,882), with no interaction between BMI subgroups (*P* = 0.58 and *P* = 0.44, respectively).^29^ Deitelzweig et al. examined electronic health record data of Veteran Affairs and Medicare NVAF obese patients, including 6112 who were severely obese (BMI ≥ 40 kg/m^2^), and they demonstrated that stroke risk in apixaban-treated patients (*n* = 13,604) was similar (HR 0.82; 95% CI 0.66-1.03) and that bleeding risk was lower (HR 0.62; 95% CI 0.54-0.70), compared with that for warfarin-treated patients (*n* = 12,918).^35^ A recent meta-analysis of subgroups of phase III RCTs, post hoc analyses of RCTs, and observational cohorts assessing the safety and effectiveness of DOACs vs warfarin in NVAF patients across BMI categories also found that obese DOAC (BMI ≥ 30 kg/m^2^) users were at similar risk for stroke/SE (HR 0.87; 95% CI 0.73-1.04) and major bleeding (HR 0.90; 95% CI 0.81-1.01).^36^ Another meta-analysis including only the 4 DOAC vs warfarin RCTs (**R**andomized **E**valuation of **L**ong-term Anticoagulation Therap**y** [RE-LY], ROCKET-AF, ARISTOTLE, and ENGAGE AF-TIMI 48) stratified by BMI also found similar efficacy and safety with DOACs vs warfarin.^37^ Despite limited data in patients with BMI ≥ 40 kg/m^2^, the safety and efficacy of apixaban and edoxaban appeared to be similar to that of warfarin in patients with a BMI of 40-50 kg/m^2^.^37^

Based on the latest evidence, an algorithm for DOAC choice according to the severity of obesity was subsequently proposed.^38^ In patients with a BMI of 40-50 kg/m^2^, they recommend use of warfarin and consideration of use of apixaban or edoxaban, based on lack of evidence for use of dabigatran and rivaroxaban in this BMI category, and on subgroup analysis with apixaban and edoxaban that does not suggest any inferior benefit, compared with warfarin.^38^ In patients with a BMI ≥ 50 kg/m^2^, they recommend use of warfarin, citing limited evidence regarding use of DOACs in this BMI category, and concerns about the impact of obesity on OAC.^38^

This analysis is the first large real-world comparison of the safety and effectiveness of DOACs vs warfarin in a Canadian population. The Canadian population differs from other populations, such as the American population, in many ways, such as the prevalence and severity of obesity,^39^^,^^40^ the racial and ethnic composition, the socioeconomic and educational distribution, and the healthcare/medication governmental coverage. Therefore, the comparative safety and effectiveness of DOACs vs warfarin might differ between the American population and populations with an obesity severity and prevalence comparable to those in the Canadian population.^39^^,^^40^ Additionally, our analysis used a province-wide single-payer Quebec healthcare claims database. Given that most important clinical events would have resulted in an administrative claim, and few patients seek medical services outside of the province, nearly all clinically significant events likely have been captured, which may not have been the case in previous single-hospital or single-insurer studies.

Some limitations must be acknowledged in interpreting the results of our analysis. First, this analysis is observational and it used administrative data that may be subject to confounding by unadjusted factors (eg, ethnicity, over-the-counter prescription use, fluctuations in BMI). Second, administrative data claims depend on complete and accurate recording of diagnoses, as well as of procedure and drug codes. Third, our results may not be generalizable to younger populations or to patients treated with other DOACs or reduced-dose rivaroxaban or apixaban. Forth, event sizes were limited for individual outcome of the composite safety and effectiveness outcomes. Fifth, time in therapeutic range could not be used to assess appropriateness of warfarin dosing, as an international normalized ratio was not available in our database. Finally, obesity was necessarily based on ICD-codes and not on BMI per se, given that weight and height are not available in claims data. Although ICD codes for obesity have a high PPV and specificity,^13^^,^^16^^,^^41^^,^^42^ some AF patients with obesity, particularly non-severe obesity, may well not have been captured in our analysis.

## Conclusion

DOACs were associated with a similar efficacy profile, compared with warfarin therapy in NVAF obese patients, and a better or similar safety profile. Future studies need to examine whether these findings are applicable to other DOACs and reduced-dose regimens in obese patients, and future RCTs should seek to include more patients with extreme BMIs.
