# Supplementary material for: Comparative Effectiveness and Safety of Direct Oral Anticoagulants vs Warfarin Among Obese Patients With Atrial Fibrillation
Source: CJC Open. 2022 Jan 13;4(4):395–405. doi: 10.1016/j.cjco.2022.01.002 (PMC9039573; doi:10.1016/j.cjco.2022.01.002)
Supplement: Supplemental Tables S1-S10 [file mmc1.pdf]

Supplemental Table S1. Definition of variables outcomes according to ICD-9 and ICD-10 from Med-Echo databases.

	ICD-9 codes	ICD-10 codes
**Thromboembolic events**		
**-Stroke**		
Hemorrhagic stroke intracranial (non-traumatic)	430, 431, 432.x	I60, I61, I62
Ischaemic stroke	433.xx, 434.xx, 436.0, 436.9 (primary diagnosis only using Med-Echo)	I63 except I63.6, I64 (primary diagnosis only using Med-Echo)
**- Systemic embolism (SE)**	444.x, 557.0, 362.31, 362.32, 598.31 (primary diagnosis only using Med-Echo)	I74 (primary only)
Arterial embolism and thrombosis	444.x (primary diagnosis only using Med-Echo)	I74.0, I74.1, I74.2, I74.3, I74.5, I74.8, I74.9 (primary diagnosis only using Med-Echo)
Ischemic colitis or mesenteric thromboembolism	557.0 (primary diagnosis only using Med-Echo)	K55.0 (primary diagnosis only using Med-Echo)
Retinal artery thromboembolism	362.31, 362.32 (primary diagnosis only using Med-Echo)	H34.1, H34.2 (primary diagnosis only using Med-Echo)
Renal artery thromboembolism	593.81 (primary diagnosis only using Med-Echo)	N28.0 (primary diagnosis only using Med-Echo)
**All-cause of deaths**		
**Myocardial Infarction**	410	I21
**Major Bleedings**		
**-Intracranial Major bleeding**		
Intracranial bleeding	430, 431, 432.x, 852.x, 853.x (primary diagnosis or the first secondary diagnosis using Med-Echo)	I60, I61, I62, S06.3, S06.4, S06.5, S06.6 (primary diagnosis or the first secondary diagnosis using Med-Echo)
**-Major GI bleeding**		
Upper gastrointestinal bleeding (only using Med-Echo)	456.1, 530.7, 531.0x, 531.2x, 531.4x, 531.6x, 532.0x, 532.2x, 532.4x. 532.6x, 533.0x. 533.2x, 533.4x, 533.6x, 534.0x, 534.2x, 534.4x, 534.6x, 535.1, 578.0 (primary diagnosis only using Med-Echo)	I85.0, K22.6, K25.0, K25.2, K25.4, K25.6, K26.0, K26.2, K26.4, K26.6, K27.0, K27.2, K27.4, K27.6, K28.0, K28.2, K28.4, K28.6, K29.0, K92.0 (primary diagnosis only using Med-Echo)
Upper gastrointestinal bleeding (only using RAMQ)	456.1, 530.7, 531.0x, 531.2x, 531.4x, 531.6x, 532.0x, 532.2x, 532.4x. 532.6x, 533.0x, 533.2x, 533.4x, 533.6x, 534.0x, 534.2x, 534.4x, 534.6x, 535.1, 578.0 RAMQ ICD-9 at an emergency room and procedure endoscopic control of gastric or duodenal bleeding or upper gastrointestinal endoscopy including esophagus, stomach, and either the duodenum and/or jejunum as appropriate with control of bleeding, any method (code 00691) within 7 days	I85.0, K22.6, K25.0, K25.2, K25.4, K25.6, K26.0, K26.2, K26.4, K26.6, K27.0, K27.2, K27.4, K27.6, K28.0, K28.2, K28.4, K28.6, K29.0, K92.0 RAMQ ICD-9 at an emergency room and procedure endoscopic control of gastric or duodenal bleeding or upper gastrointestinal endoscopy including esophagus, stomach, and either the duodenum and/or jejunum as appropriate with control of bleeding, any method (00691) within 7 days
Lower gastrointestinal bleeding	562.02, 562.03, 562.12, 562.13, 569.3x, 569.85, 578.1x, 578.9 (primary diagnosis only using Med-Echo)	K57.11, K57.13, K57.31, K57.33, K62.5, K55.21, K92.1, K92.2 (primary diagnosis only using Med-Echo)
**-Other Critical Sites of Major Bleedings**		
Gross hematuria	599.7 (primary diagnosis only using Med-Echo)	R31 (primary diagnosis only using Med-Echo)
Hemoptysis	786.3x (primary diagnosis only using Med-Echo)	R04.2, R04.89, R04.9 (primary diagnosis only using Med-Echo)
Vitreous hemorrhage	379.23 (primary diagnosis only using Med-Echo)	H43.13 (primary diagnosis only using Med-Echo)
Urogenital bleed	626.2x and 280.0 (primary only), 285.1 (principal diagnosis or the first secondary diagnosis) or 285.9 (principal diagnosis or the first secondary diagnosis using Med-Echo)	N92.0 and D50.0 (primary only), D62 (principal diagnosis or the first secondary diagnosis), D64.9 (principal diagnosis or the first secondary diagnosis using Med-Echo)
Hemarthrosis	719.1x (primary diagnosis only using Med-Echo)	M25.0x (primary diagnosis only using Med-Echo)
Hemopericardium	423.0 (primary diagnosis only using Med-Echo)	I31.2 (primary diagnosis only using Med-Echo)
Hemoperitoneum	568.8 (primary diagnosis only using Med-Echo)	K66.1 (primary diagnosis only using Med-Echo)
Hemorrhage not specified	459.0x (primary diagnosis only using Med-Echo)	R58.0 (primary diagnosis only using Med-Echo)
Acute posthemorrhagic anemia	285.1x (primary diagnosis only using Med-Echo)	D62 (primary diagnosis only using Med-Echo)

Supplemental Table S2. Risk score definition for CHADS2, CHA_2DS_2-Vasc and Modified HAS-BLED.

**Risk score definition**	Points if present
**CHADS-2**	
Congestive heart failure or left ventricular dysfunction	1
Hypertension	1
Age ≥ 75 years	1
Diabetes Mellitus	1
Stroke (ischemic stroke, transient ischemic disease or systemic embolism)	2
**CHA_2DS_2-VASc**	
Congestive heart failure or left ventricular dysfunction	1
Hypertension	1
Age 65 – 74 years	1
Age ≥ 75 years	2
Diabetes Mellitus	1
Stroke (ischemic stroke, transient ischemic disease or systemic embolism	2
Vascular disease (myocardial infarction, peripheral arterial disease or aortic plaque	1
Sex category (female)	1
**HAS-BLED**	
Hypertension	1
Abnormal renal function	1
Abnormal hepatic function	1
Abnormal Stroke (ischemic stroke, transient ischemic disease	1
Bleeding	1
Older than > 65 years	1
Labile international normalized ratio (not available)	1
Drugs (ASA, clopidogrel, prasugrel, ticagrelor, ticlopidine, or non-steroidal anti-inflammatory drugs) in the 1 month preceding the ICH hospitalization or 1month after discharge	1
Alcohol intake	1

Supplemental Table S3. Definition of variables used in the risk score definition of $CHADS_2$ according to ICD-9 and ICD-10 from Med-Echo databases.

	ICD-9 codes	ICD-10 codes
**$CHADS_2$**		
Congestive heart failure	402.01, 402.11, 402.91, 404.01, 404.11, 404.91, 404.03, 404.13, 404.93, 425.4, 428.0	I11.0, I13.0, I13.2, I42.0, I50
Left ventricular dysfunction	428.1, 428.9	I50.1, I50.9
Hypertension	401	I10
Diabetes	250.x	E08, E10, E11, E13
Ischemic stroke	433.xx, 434.xx, 436	I63 except 63.6, I67.89
Systemic embolism	444.x, 557.0, 362.31, 362.32, 598.31	I74, K55.0, H34.1, H34.2, N28.0
Transient ischemic stroke (TIA)	435.x	G45

Supplemental Table S4. Definition of variables used in the risk score definition of CHA_2DS_2-VASc according to ICD-9 and ICD-10 from Med-Echo databases.

	ICD-9 codes	ICD-10 codes
**CHA_2DS_2-VASc**		
Congestive heart failure	402.01, 402.11, 402.91, 404.01, 404.11, 404.91, 404.03, 404.13, 404.93, 425.4, 428.0	I11.0, I13.0, I13.2, I42.0, I50
Left ventricular dysfunction	428.1, 428.9	I50.1, I50.9
Hypertension	401	I10
Diabetes	250.x	E08, E10, E11, E13
Ischemic stroke	433.xx, 434.xx, 436	I63 except 63.6, I67.89
Systemic embolism	444.x, 557.0, 362.31, 362.32, 598.31	I74, K55.0, H34.1, H34.2, N28.0
Transient ischemic stroke (TIA)	435.x	G45
Aortic plaque	440.0	I70.0
Peripheral arterial disease	440 (except 440.0), 441, 443.0, 443.89, 443.9	I70.1 to I70.9, I71, I73.0, I73.89, I73.9
Myocardial infarction	410.xx	I21, I22, I23

Supplemental Table S5. Definition of variables used in the risk score definition of HAS-BLED based on associated morbidities and concomitant drug.

	ICD-9	ICD-10
**Modified HASBLEED**		
Ischemic stroke	433.xx, 434.xx, 436	I63 except I63.6, I67.89
Transient ischemic-attack	435.x	G45
Moderate to severe renal disease	404.01, 404.03, 404.11, 404.13, 404.91, 404.93, 580.0, 580.4, 581.0, 581.1, 581.2, 581.3, 581.89, 581.9, 582.0, 582.1, 582.2, 582.89, 582.9, 583.0, 583.1, 583.2, 583.4, 583.7, 583.6, 583.89, 583.9, 584.5, 584.6, 584.7, 584.8, 584.9, 585.1, 585.2, 585.3, 585.4, 585.5, 585.6, 586, 590.0, 590.01, 590.80	I12, I13, N00, N01, N02, N03, N04, N05, N07, N11, N12, N14, N17, N18, N19
Moderate to severe liver disease	570, 572.3, 070.0, 070.21, 070.20, 070.60	K7200, K762, K766, B150, B160, B162, B190, K704, I85
Haemorrhagic stroke intracranial (non-traumatic)	430, 431, 432.x	I60, I61, I62
Extracranial major or unclassified major bleeding	Upper GI: 456.1, 530.7, 531.0x, 531.2x, 531.4x, 531.6x, 532.0x, 532.2x, 532.4x. 532.6x, 533.0x. 533.2x, 533.4x, 533.6x, 534.0x, 534.2x, 534.4x, 534.6x, 535.01, 537.83, 578.0<br>Lower GI: 562.02, 562.03, 562.12, 562.13, 569.3x, 569.85, 578.1x, 578.9<br>Other sites:<br>626.2x and 280.0, 285.1 or 285.9<br>599.7, 786.3x, 379.23, 719.1x, 423.0x, 568.8, 459.0x, 285.1x	Upper GI: I850, K226, K250, K252, K254, K256, K260, K262, K264, K266, K270, K272, K274, K276, K280, K282, K284, K286, K2901, K290, K31811, K920<br><br>Lower GI: K921, K922, K5711, K5713, K5731, K5733, K625, K5521<br>Other sites:<br>N92.0 and D50.0 or D62 or D64.9,<br>R31, R042, R0489, R049, H43.13,<br>M250x, I31.2, K66.1, R58.0, D62
Gastrointestinal bleeding	Upper GI: 531.0x, 531.2x, 531.4x, 531.6x, 532.0x, 532.2x, 532.4x. 532.6x, 533.0x. 533.2x, 533.4x, 533.6x, 534.0x, 534.2x, 534.4x, 534.6x, 535.01, 537.83, 578.0<br>Lower GI: 562.02, 562.03, 562.12, 562.13, 569.3x, 569.85, 578.1x, 578.9	Upper GI: K250, K252, K254, K256, K260, K262, K264, K266, K270, K272, K274, K276, K280, K282, K284, K286, K2901, K290, K31811, K920<br>Lower GI: K921, K922, K5711, K5713, K5731, K5733, K625, K5521
Traumatic intracranial bleeding	852x, 853x	S063, S064, S065, S066
Clopidogrel, ticlopidine, prasugrel, ticagrelor	46486, 47307, 45617, 47402, 47834, 47866	46486, 47307, 45617, 47402, 47834, 47866
Low dose ASA	00143, 46353 (daily dose < 100 mg)	00143, 46353 (daily dose < 100 mg)
Non-steroidal anti-inflammatory drugs (NSAIDs)	46353, 38184, 47327, 47078, 41694, 47059, 43150, 47122, 33803, 44749, 04745, 46654, 47506, 04810, 38691, 44359, 47385, 47084, 19752, 47890, 07462, 42019, 47346, 47107, 40381, 45592, 45407, 03766	46353, 38184, 47327, 47078, 41694, 47059, 43150, 47122, 33803, 44749, 04745, 46654, 47506, 04810, 38691, 44359, 47385, 47084, 19752, 47890, 07462, 42019, 47346, 47107, 40381, 45592, 45407, 03766
Alcohol	331.7, 359.4, 425.5, 577.1	E224, E529A, F10, G312, G612, G721, I426, K292, K70, K860, L278A, O354, T51, Z714, Z721

Supplemental Table S6. Demographic and clinical characteristics of new user of high dose of DOAC and warfarin from 2011 to 2017.

	Warfarin (n=1 253)	Rivaroxaban 20 mg† (n=403)	Rivaroxaban 15 mg† (n=77)	Apixaban 5 mg* (n=539)	Apixaban 2,5 mg* (n=101)	Dabigatran 150 mg* (n=150)	Dabigatran 110 mg* (n=100)
Age (mean ± SD‡)	74.54 ± 9.80	69.30 ± 8.76	79.49 ± 8.11	73.50 ± 8.63	83.64 ± 6.86	67.29 ± 7.90	76.74 ± 7.18
Male (n, %)	522 (41.66)	194 (48.14)	25 (32.47)	258 (47.87)	32 (31.68)	83 (55.33)	51 (51.00)
CHADS-2 Score (mean ± SD)	2.78 ± 1.26	2.10 ± 1.22	3.00 ± 1.18	2.48 ± 1.20	3.19 ± .124	2.08 ±1.11	2.60 ±1.18
Score 0 – 1 (n, %)	177 (14.13)	125 (33.50)	7 (9.09)	115 (21.34)	6 (5.94)	46 (30.67)	16 (16.00)
Score 2 – 3 (n, %)	754 (60.18)	216 (33.50)	48 (62.34)	326 (60.48)	59 (58.42)	88 (58.67)	64 (64.00)
Score ≥ 4 (n, %)	322 (25.70)	52 (12.90)	22 (28.57)	98 (18.18)	36 (35.64)	16 (10.67)	20 (20.00)
CHA_2DS_2-VASc (mean ± SD)	3.55 ± 1.40	2.81 ± 1.39	3.74 ± 1.32	3.10 ± 1.36	3.78 ± 1.30	2.70 ± 1.30	3.53 ± 1.28
Score 0 – 1 (n, %)	85 (6.78)	58 (14.39)	2 (2.60)	48 (8.91)	(0.99)	33 (22.00)	2 (2.00)
Score 2 – 3 (n, %)	490 (39.11)	223 (55.33)	20 (25.97)	262 (48.61)	21 (20.79)	82 (54.67)	54 (54.00)
Score ≥ 4 (n, %)	678 (54.11)	122 (30.27)	55 (71.43)	229 (42.29)	79 (78.22)	35 (23.33)	44 (44.00)
HAS-BLED score (mean ± SD)	3.30 ± 1.32	2.61 ± 1.28	3.47 ± 1.15	2.90 ± 1.26	3.58 ± 1.27	2.43 ± 1.25	3.28 ± 1.09
Score < 3 (n, %)	313 (24.98)	190 (47.15)	15 (19.48%)	198 (36.73)	20 (19.80)	85 (56.67)	25 (25.00)
Score ≥ 3 (n, %)	940 (75.02)	213 (52.85)	62 (80.52%)	341 (62.27)	81 (80.20)	65 (43.33)	75 (75.00)
Charlson score (mean, ± SD)	5.21 ± 3.25	4.13 ± 3.26	5.97 ±3.88	4.47 ± 3.34	5.84 ± 3.22	3.91 ± 3.01	4.49 ± 3.26
Score < 4 (n, %)	414 (33.04)	205 (50.87)	22 (28.57)	240 (44.54)	26 (25.74)	84 (56.00)	42 (42.00)
Score ≥ 4 (n, %)	839 (66.96)	198 (49.13)	299 (55.47)	299 (55.47)	75 (74.26)	66 (44.00)	58 (58.00)
**Comorbidities including index hospitalization and 3-year prior index**							
Hypertension (n, %)	1118 (89.23)	337 (83.63)	71 (92.21)	478 (88.68)	92 (91.09)	121 (80.67)	91 (91.00)
Coronary artery disease (n, %)	760 (60.65)	185 (45.91)	48 (62.34)	256 (47.50)	72 (71.29)	79 (52.67)	63 (63.00)
Acute myocardial infarction (n, %)	168 (13.41)	43 (10.67)	8 (10.39)	53 (9.83)	20 (19.80)	12 (8.00)	13 (13.00)
Chronic heart failure (n, %)	590 (47.09)	129 (32.01)	42 (54.55)	213 (39.52)	59 (58.42)	52 (34.67)	38 (38.00)
Valvular heart disease (n, %)	260 (20.75)	47 (11.66)	17 (22.08)	76 (14.10)	25 (24.75)	134 (89.33)	16 (16.00)
Cardiomyopathy (n, %)	96 (7.66)	30 (7.44)	7 (9.09)	41 (7.61)	5 (4.95)	9 (6.00)	6 (6.00)
Other cardiac dysrhythmias (n, %)	232 (18.52)	66 (16.38)	16 (20.78)	91 (16.88)	18 (17.82)	25 (16.67)	23 (23.00)
Peripheral arterial disease (n, %)	287 (22.91)	61 (15.14	17 (22.08)	72 (13.36)	30 (29.70)	21 (14.00)	100 (3.81)
Dyslipidemia (n, %)	778 (62.09)	238 (59.06)	48 (62.34)	317 (58.81)	74 (73.27)	82 (54.67)	62 (62.00)
Diabetes (n, %)	789 (62.97)	208 (51.61)	39 (50.65)	305 (56.59)	53 (52.45)	80 (53.33)	52 (52.00)
Major bleeding (n, %)							
Major intracranial bleeding	27 (2.15)	8 (1.99)	3 (3.90)	19 (3.53)	4 (3.96)	1 (0.67)	3 (3.00)
Major GI bleeding	100 (7.98)	18 (4.47)	10 (12.99)	29 (5.38)	10 (9.90)	8 (5.33)	14 (14.00)
Other major sites of major bleeding	335 (26.74)	63 (15.63)	27 (35.06)	95 (17.63)	27 (26.73)	15 (10.00)	32 (32.00)
Chronic renal failure (n, %)	623 (49.72)	94 (23.33)	45 (58.44)	68 (31.17)	65 (64.36)	30 (20.00)	32 (32.00)
…Chronic renal failure ≤ 30 mL/min	145 (11.57)	7 (0.74)	7 (9.09)	12 (2.23)	6 (5.94)	5 (3.33)	4 (4.00)
Acute renal failure (n, %)	435 (34.72)	62 (15.38)	30 (38.96)	118 (21.89)	47 (46.53)	16 (10.67)	23 (23.00)
Liver disease (n, %)	31 (2.47)	12 (2.98)	1 (1.30)	18 (3.34)	4 (3.96)	3 (2.00)	4 (4.00)
Chronic obstructive pulmonary disease (n, %)	582 (46.46)	190 (47.15)	43 (55.84)	234 (43.31)	54 (53.47)	65 (43.33)	49 (49.00)
Systemic embolism (n, %)	36 (2.87)	5 (1.24)	0 (0.00)	10 (1.86)	3 92.97)	2 (1.33)	1 (1.00)
Helicobacter Pylori infection (n, %)	13 (1.04)	1 (0.25)	0 (0.00)	3 (0.56)	2 (1.98)	1 (0.67)	0 (0.00)
Depression (n, %)	142 (11.41)	50 (12.41)	11 (14.29)	60 (11.13	14 (13.86)	15 (10.00)	11 (11.00)
Hypothyroidism (n, %)	297 (23.70)	82 (20.35)	24 (31.17)	94 (17.44)	33 (32.67)	22 (14.67)	17 (17.00)

Neurological disorder (n, %)	257 (20.51)	67 (16.63)	18 (23.38)	93 (17.25)	39 (38.61)	15 (10.00)	21 (21.00)
Malign cancer (n, %)	235 (18.75)	88 (21.84)	23 (29.87)	117 (21.71)	22 (21.78)	27 (18.00)	21 (21.00)
**Medical procedures (3-year prior to entry)**							
Cardiac catheterization (n, %)	72 (5.83)	21 (5.21)	6 (7.79)	28 (5.19)	5 90.19)	8 (5.33)	7 (7.00)
Percutaneous coronary intervention – Stent (n, %)	48 (3.83)	12 (2.98)	2 (2.60)	12 (2.23)	4 (3.96)	7 (4.67)	3 (3.00)
Coronary artery bypass grafting (n, %)	19 (1.52)	6 (1.49)	0 (0.00)	13 (2.41)	1 (0.99)	6 (4.00)	2 (2.00)
Medical procedures for cerebrovascular disease (n, %)	16 (1.28)	2 (0.50)	0 (0.00)	1 (0.19)	0 (0.00)	0 (0.00)	1 (1.00)
Implantable cardiac devices (n,%)	12 (0.96)	0 (0.00)	0 (0.00)	0 (0.00)	0 (0.00)	0 (0.00)	0 (0.00)
**Medications (3-month prior to cohort entry)**							
Statin (n, %)	872 (69.59)	268 (66.50)	57 (74.03)	365 (67.72)	71 (70.30)	101 (67.33)	72 (72.00)
Antiplatelet (excluding low dose ASA) (n, %)	169 (13.49)	33 (8.19)	12 (4.11)	38 (7.05)	18 (17.82)	11 (7.33)	11 911.00)
Low dose ASA (n, %)	810 (64.64)	199 (49.38)	47 (61.04)	273 (50.65)	56 (55.45)	77 (51.33)	60 (60.00)
Proton pump inhibitors (PPIs) (n, %)	757 (60.42)	204 (50.62)	49 (63.64)	288 (53.43)	71 (70.30)	65 (43.33)	58 (58.00)
NSAIDs (n, %)	89 (7.10)	35 (8.68)	7 (9.09)	47 (8.72)	3 (2.97)	19 (0.72)	10 (10.00)
Digoxin (n, %)	177 (14.13)	38 (9.43)	10 (12.99)	58 (10.76)	12 (11.88%)	16 (10.67)	18 (18.00)
Amiodarone (n, %)	130 (10.38)	34 (8.44)	7 (9.09)	59 (10.95)	10 (9.90)	13 (8.67)	16 (16.00)
Antidepressants (n, %)	140 (11.17)	45 (11.17)	14 (18.18)	62 (11.50)	16 (15.84)	19 (12.67)	15 (15.00)
B-Blockers (n, %)	883 (70.47)	294 (72.95)	52 (67.53)	381 (70.69)	78 (77.23)	114 (76.00)	62 (62.00)
Calcium channel blockers (n, %)	737 (58.82)	206 (51.12)	50 (51.12)	280 (51.95)	58 (57.43)	74 (49.33)	57 (57.00)
Inhibitors of renin-angiotensin system (n, %)	781 (62.33)	224 (55.58)	44 (57.14)	321 (59.55)	59 (58.42)	89 (59.33)	72 (72.00)
Loop diuretics (n, %)	679 (54.19)	138 (32.24)	41 (53.25)	243 (43.31)	66 (65.35)	50 (33.33)	44 (44.00)
Diuretics (n, %)	837 (66.80)	191 (47.39)	48 (62.34)	294 (54.55)	76 (75.25)	83 (55.33)	57 (57.00)
Antidiabetics (n, %)	659 (52.59)	174 (43.18)	29 (37.66)	258 (47.87)	44 (43.56)	71 (47.33)	42 (42.00)
**Medications (Initiation or 2 weeks prior entry)**							
Statin (n, %)	685 (52.48)	205 (50.87)	39 (50.65)	257 (47.68)	48 (47.52)	73 (48.67)	51 (51.00)
Antiplatelet (excluding low dose ASA)[¥] (n, %)	82 (6.54)	17 (4.22)	9 (11.69)	16 (2.97)	9 (8.91)	5 (3.33)	4 (4.00)
Low dose ASA[¥] (n, %)	473 (37.75)	105 (26.05)	26 (33.77)	123 (22.82)	28 (27.72)	41 (27.33)	36 (36.00)
Proton pump inhibitors (PPIs) (n, %)	581 (46.37)	130 (32.26)	35 (45.45)	190 (35.25)	42 (41.58)	50 (33.33)	43 (43.00)
NSAIDs[€] (n, %)	29 (2.31)	9 (2.23)	1 (1.30)	8 (1.48)	1 (0.99)	8 (5.33)	1 (1.00)
Digoxin (n, %)	171 (13.65)	36 (8.93)	10 (12.99)	58 (10.76)	10 (9.90)	14 (9.33)	16 (16.00)
Amiodarone (n, %)	126 (10.06)	34 (8.44)	6 (7.79)	56 (10.39)	10 (9.90)	13 (8.67)	14 (14.00)
Antidepressants (n, %)	116 (9.26)	30 (7.44)	10 (12.99)	45 (8.35)	14 (13.86)	11 (7.33)	12 (12.00)
B-Blockers (n, %)	74 (59.54)	251 (62.28)	42 (54.55)	336 (62.34)	64 (63.37)	93 (62.00)	47 (47.00)
Calcium channel blockers (n, %)	530 (42.30)	162 (40.20)	35 (45.45)	190 (35.25)	35 (34.65)	44 (29.33)	39 (39.00)
Inhibitors of renin-angiotensin system (n, %)	548 (43.74)	164 (40.69)	30 (38.96)	230 (42.67)	38 (37.62)	66 (44.00)	48 (48.00)
Loop diuretics (n, %)	575 (45.89)	120 (29.78)	35 (45.45)	208 (38.59)	56 (55.45)	45 (30.00)	40 (40.00)
Diuretics (n, %)	670 (53.47)	154 (38.21)	42 (54.55)	247 (45.83)	62 (61.39%)	70 (46.67)	46 (46.00)

Antidiabetic (n,%)	505 (40.30)	130 (32.26)	20 (25.97)	194 (35.99)	33 (32.67)	56 (37.33)	33 (33.00)
**Medical service (1-year prior to entry)**							
Number of specialty visits (mean ± SD‡)	3.20 ± 4.61	3.00 ±5.32	3.37 ± 4.76	3.56 ± 4.95	3.09 ± 4.10	3.28 ± 6.11	4.09 ± 5.36
Number of family physician visits (mean ± SD‡)	4.46 ± 8.37	2.65 ± 5.69	3.51 ± 5.83	2.42 ± 5.35	5.27 ±9.71	3.62 ±6.48	6.01 ±13.47
**Hospital service (3-year prior to entry)**							
Number of emergency visits (mean ± SD‡)	5.23 ± 4.86	4.46 ± 4.10	6.06 ± 5.69	4.74 ± 5.85	6.51 ± 6.79	4.04 ± 3.81	4.72 ± 4.44
≥2 all-cause hospital admission (n, %)	729 (59.78)	198 (49.13)	52 (67.53)	275 (51.02)	57 (56.44)	65 (43.33)	61 (61.00)
Number of all-cause hospital admission (mean ±SD‡)	2.46 ± 1.96	2.07 ± 1.59	2.51 ± 1.58	2.20 ± 2.05	2.56 ± 2.39	1.87 ± 1.49	2.31 ± 1.78

*: BID: Twice daily; †: DIE: Once daily; ‡: SD: Standard deviation; ¥: Acetylsalicylic acid: ASA; €: Nonsteroidal anti-inflammatory drugs: NSAIDs.

Supplemental Table S7. Cohort using inverse-probability-of-treatment-weighting of high-dose rivaroxaban vs warfarin.

	Initial cohort			Cohort using IPTW		
	Warfarin (n=1 253)	Rivaroxaban 20 mg† (n=403)	Absolute standardized difference	Warfarin (n=1,253)	Rivaroxaban 20 mg† (n=403)	Absolute standardized difference
Age (mean ± SD‡)	74.54 ± 9.80	69.30 ± 8.76	0.60	72.83 ± 11.07	71.91 ± 8.09	0.11
Male (n, %)	522 (41.66)	194 (48.14)	0.13	551 (43.71)	178 (45.57)	0.04
CHADS-2 Score (mean, ± SD)	2.78 ± 1.26	2.10 ± 1.22	0.55	2.60 ± 1.28	2.60 ± 1.28	0.00
HAS-BLED score (mean ± SD)	3.54 ± 1.40	2.81 ± 1.39	0.53	3.35 ± 1.44	3.35 ± 1.43	0.00
Charlson score (mean, ± SD)	5.21 ± 3.25	4.13 ± 3.26	0.33	4.92 ± 3.30	5.11 ± 3.35	0.06
**Comorbidities including index hospitalization and 3-year prior index**						
Hypertension (n, %)	1118 (89.23)	337 (83.63)	0.16	1100 (87.28)	344 (88.06)	0.02
Coronary artery disease (n, %)	760 (60.65)	185 (45.91)	0.30	710 (56.35)	217 (55.48)	0.02
Acute myocardial infarction (n, %)	168 (13.41)	43 (10.67)	0.08	157 (12.45)	45 (11.51)	0.03
Chronic heart failure (n, %)	590 (47.09)	129 (32.01)	0.31	544 (43.12)	174 (33.52)	0.03
Valvular heart disease (n, %)	260 (20.75)	47 (11.66)	0.25	237 (18.82)	75 (19.30)	0.01
Cardiomyopathy (n, %)	96 (7.66)	30 (7.44)	0.01	93 (7.39)	26 (6.65)	0.03
Other cardiac dysrhythmias (n, %)	232 (18.52)	66 (16.38)	0.06	225 (17.83)	75 (19.20)	0.04
Peripheral arterial disease (n, %)	287 (22.91)	61 (15.14	0.20	263 (20.85)	88 (22.57)	0.04
Dyslipidemia (n, %)	778 (62.09)	238 (59.06)	0.06	772 (61.20)	243 (62.09)	0.02
Diabetes (n, %)	789 (62.97)	208 (51.61)	0.23	756 (59.93))	237 (60.72)	0.02
Major bleeding (n, %)	842 (67.20)	322 (79.90)	0.29	372 (29.50)	112 (28.54)	0.02
Chronic renal failure (n, %)	623 (49.72)	94 (23.33)	0.57	540 (42.85)	163 (41.72)	0.02
Acute renal failure (n, %)	435 (34.72)	62 (15.38)	0.46	5375 (29.76)	111 (28.40)	0.03
Liver disease (n, %)	31 (2.47)	12 (2.98)	0.03	32 (2.55)	12 (2.97)	0.03
Chronic obstructive pulmonary disease (n, %)	582 (46.46)	190 (47.15)	0.01	587 (46.58))	194 (49.63)	0.06
Systemic embolism (n, %)	36 (2.87)	5 (1.24)	0.12	32 (2.51)	11 (2.81)	0.02
Helicobacter Pylori infection (n, %)	13 (1.04)	1 (0.25)	0.10	11 (0.85)	5 (1.18)	0.03
Depression (n, %)	142 (11.41)	50 (12.41)	0.03	150 (11.87)	49 (12.54)	0.02
Hypothyroidism (n, %)	297 (23.70)	82 (20.35)	0.08	281 (22.31)	78 (20.05)	0.06
Neurological disorder (n, %)	257 (20.51)	67 (16.63)	0.10	243 (19.27)	70 (17.88)	0.04
Malign cancer (n, %)	235 (18.75)	88 (21.84)	0.08	244 (19.34)	82 (21.10)	0.04
**Medical procedures (3-year prior to entry)**						
Cardiac catheterization (n, %)	72 (5.83)	21 (5.21)	0.03	70.16 (5.57)	25 (6.37)	0.03
Percutaneous coronary intervention – Stent (n, %)	48 (3.83)	12 (2.98)	0.05	45 (3.59)	15 (3.80)	0.01
Coronary artery bypass grafting (n, %)	19 (1.52)	6 (1.49)	0.00	18 (1.45)	5 (1.39)	0.01
Implantable cardiac devices (n, %)	12 (0.96)	0 (0.00)	.014	9 (0.72)	0 (0.00)	0.12
**Medications (Initiation or 2 weeks prior entry)**						
Statin (n, %)	685 (52.48)	205 (50.87)	0.08	675 (53.52))	224 (57.31)	0.08
Antiplatelet (excluding low dose ASA)¥ (n, %)	82 (6.54)	17 (4.22)	0.10	75 (5.94)	28 (7.14)	0.05
Low dose ASA¥ (n, %)	473 (37.75)	105 (26.05)	0.25	441 (34.97)	146 (37.48)	0.05

Proton pump inhibitors (PPIs) (n, %)	581 (46.37)	130 (32.26)	0.29	536 (42.50)	159 (40.78)	0.03
NSAIDs€ (n, %)	29 (2.31)	9 (2.23)	0.01	30 (2.40)	17 (4.30)	0.11
Digoxin (n, %)	171 (13.65)	36 (8.93)	0.15	157 (12.44)	47 (11.96)	0.01
Amiodarone (n, %)	126 (10.06)	34 (8.44)	0.06	122 (9.64)	34 (8.64)	0.04
Antidepressants (n, %)	116 (9.26)	30 (7.44)	0.07	110 (8.76)	34 (8.62)	0.01
B-Blockers (n, %)	74 (59.54)	251 (62.28)	0.06	760 (60.30))	236 (60.42)	0.00
Calcium channel blockers (n, %)	530 (42.30)	162 (40.20)	0.04	525 (41.67)	164 (42.06)	0.01
Inhibitors of renin-angiotensin system (n, %)	548 (43.74)	164 (40.69)	0.06	540 (42.80)	181 (46.22)	0.07
Loop diuretics (n, %)	575 (45.89)	120 (29.78)	0.34	526 (41.69)	166 (42.49)	0.02
Diuretics (n, %)	670 (53.47)	154 (38.21)	0.31	621 (49.30)	194 (49.68)	0.01
Antidiabetics (n, %)	505 (40.30)	130 (32.26)	0.17	483 (38.31)	149 (39.09)	0.00
**Medical service (1-year prior to entry)**						
Number of specialty visits (mean ± SD‡)	3.20 ± 4.61	3.00 ±5.32	0.04	1.11 ± 2.05	1.03 ± 2.59	0.03
Number of family physician visits (mean ± SD‡)	4.46 ± 8.37	2.65 ± 5.69	0.14	1.30 ± 2.78	1.34 ± 3.61	0.01
**Hospital service (3-year prior to entry)**						
Number of emergency visits (mean ± SD‡)	5.23 ± 4.86	4.46 ± 4.10	0.17	3.22 ± 2.73	3.13 ± 2.50	0.04
Number of all-cause hospital admission (mean ±SD‡)	2.46 ± 1.96	2.07 ± 1.59	0.22	2.35 ± 1.91	2.44 ± 1.79	0.05

†: DIE: Once daily; ‡: SD: Standard deviation; ¥: Acetylsalicylic acid: ASA; €: IPTW: Inverse-probability-treatment-weighting: Nonsteroidal anti-inflammatory drugs: NSAIDs.

Supplemental Table S8. Cohort using inverse-probability-of-treatment-weighting of high-dose apixaban vs warfarin.

	Initial cohort			Cohort using IPTW		
	Warfarin (n=1 253)	Apixaban* 5 mg BID (n=539)	Absolute standardized difference	Warfarin (n=1,253)	Apixaban* 5 mg BID (n=539)	Absolute standardized difference
Age (mean ± SD‡)	74.54 ± 9.80	73.50 ± 8.63	0.12	74.25 ± 9.87	74.22 ± 8.26	0.00
Male (n, %)	522 (41.66)	258 (47.87)	0.13	547 (43.68)	239 (44.45)	0.02
CHADS-2 Score (mean, ± SD)	2.78 ± 1.26	2.48 ± 1.20	0.24	2.69 ± 1.25	2.71 ± 1.22	0.01
HAS-BLED score (mean ± SD)	3.54 ± 1.49	3.10 ± 1.36	0.33	3.42 ± 1.38	3.50 ± 1.48	0.05
Charlson score (mean, ± SD)	5.21 ± 3.25	4.47 ± 3.34	0.22	5.00 ± 3.30	5.15 ± 3.49	0.04
**Comorbidities including index hospitalization and 3-year prior index**						
Hypertension (n, %)	1118 (89.23)	478 (88.68)	0.01	1119 (89.26)	485 (90.22)	0.03
Coronary artery disease (n, %)	760 (60.65)	256 (47.50)	0.27	710 (56.63)	304 (56.51)	0.00
Acute myocardial infarction (n, %)	168 (13.41)	53 (9.83)	0.11	158 (12.59)	85 (15.75)	0.09
Chronic heart failure (n, %)	590 (47.09)	213 (39.52)	0.15	561 (44.69)	235 (43.71)	0.02
Valvular heart disease (n, %)	260 (20.75)	76 (14.10)	0.18	234 (18.62)	97 (18.10)	0.01
Cardiomyopathy (n, %)	96 (7.66)	41 (7.61)	0.00	94 (7.47)	36 (6.76)	0.03
Other cardiac dysrhythmias (n, %)	232 (18.52)	91 (16.88)	0.04	229 (18.27)	113 (21.01)	0.07
Peripheral arterial disease (n, %)	287 (22.91)	72 (13.36)	0.25	250 (29.96)	207 (20.00)	0.00
Dyslipidemia (n, %)	778 (62.09)	317 (58.81)	0.07	766 (61.06)	333 (61.99)	0.02
Diabetes (n, %)	789 (62.97)	305 (56.59)	0.13	766 (61.09)	334 (62.26)	0.02
Major bleeding (n, %)	842 (32.80)	127 (23.56)	0.21	378 (30.17)	174 (32.36)	0.05
Chronic renal failure (n, %)	623 (49.72)	68 (31.17)	0.38	555 (44.28)	244 (45.40)	0.02
Acute renal failure (n, %)	435 (34.72)	118 (21.89)	0.29	387 (30.84)	173 (32.38)	0.03
Liver disease (n, %)	31 (2.47)	18 (3.34)	0.05	34 92.74)	15 (2.73)	0.00
Chronic obstructive pulmonary disease (n, %)	582 (46.46)	234 (43.31)	0.06	568 (45.31)	254 (47.24)	0.04
Systemic embolism (n, %)	36 (2.87)	10 (1.86)	0.07	33 (2.62)	15 (2.86)	0.01
Helicobacter Pylori infection (n, %)	13 (1.04)	3 (0.56)	0.05	12 (0.92)	6 (1.13)	0.02
Depression (n, %)	142 (11.41)	60 (11.13	0.01	141 (11.24)	71 (13.21)	0.06
Hypothyroidism (n, %)	297 (23.70)	94 (17.44)	0.16	276 (22.01)	130 (24.24)	0.05
Neurological disorder (n, %)	257 (20.51)	93 (17.25)	0.08	244 (19.46)	100 (18.60)	0.02
Malign cancer (n, %)	235 (18.75)	117 (21.71)	0.07	247 (19.67)	107 (19.86)	0.00
**Medical procedures (3-year prior to entry)**						
Cardiac catheterization (n, %)	72 (5.83)	28 (5.19)	0.03	72 (5.75)	36 (6.61)	0.04
Percutaneous coronary intervention – Stent (n, %)	48 (3.83)	12 (2.23)	0.09	42 (3.32)	19 (3.56)	0.01
Coronary artery bypass grafting (n, %)	19 (1.52)	13 (2.41)	0.06	23 (1.83)	10 (1.87)	0.00
Implantable cardiac devices (n,%)	12 (0.96)	0 (0.00)	0.14	8.39 (0.67)	0 (0.00)	0.11
**Medications (Initiation or 2 weeks prior entry)**						
Statin (n, %)	685 (52.48)	257 (47.68)	0.14	662 (52.76)	291 (54.25)	0.03
Antiplatelet (excluding low dose ASA)¥ (n, %)	82 (6.54)	16 (2.97)	0.17	68 (5.45)	28 (5.26)	0.01
Low dose ASA¥ (n, %)	473 (37.75)	123 (22.82)	0.33	417 (33.24)	178 (33.09)	0.00

Proton pump inhibitors (PPIs) (n, %)	581 (46.37)	190 (35.25)	0.23	537 (42.78)	233 (43.43)	0.01
NSAIDs[€] (n, %)	29 (2.31)	8 (1.48)	0.06	26 (2.07)	10 (1.90)	0.01
Digoxin (n, %)	171 (13.65)	58 (10.76)	0.09	158 (12.57)	59 (10.95)	0.05
Amiodarone (n, %)	126 (10.06)	56 (10.39)	0.01	126 (10.08)	58 (10.87)	0.03
Antidepressants (n, %)	116 (9.26)	45 (8.35)	0.03	112 (8.94)	57 (10.61)	0.06
B-Blockers (n, %)	74 (59.54)	336 (62.34)	0.06	755 (60.21)	320 (59.62)	0.01
Calcium channel blockers (n, %)	530 (42.30)	190 (35.25)	0.15	507 (40.43)	224 (41.67)	0.03
Inhibitors of renin-angiotensin system (n, %)	548 (43.74)	230 (42.67)	0.02	548 (43.70)	231 (42.98)	0.01
Loop diuretics (n, %)	575 (45.89)	208 (38.59)	0.15	543 (43.32)	225 (41.95)	0.03
Diuretics (n, %)	670 (53.47)	247 (45.83)	0.15	637 (50.82)	271 (50.42)	0.01
Antidiabetics (n, %)	505 (40.30)	194 (35.99)	0.09	492 (39.21)	227 (42.32)	0.06
**Medical service (1-year prior to entry)**						
Number of specialty visits (mean ± SD[‡])	3.20 ± 4.61	3.56 ± 4.95	0.09	1.16 ± 2.05	1.22 ± 2.08	0.03
Number of family physician visits (mean ± SD[‡])	4.46 ± 8.37	2.42 ± 5.35	0.22	1.22 ± 2.71	1.52 ± 4.05	0.09
**Hospital service (3-year prior to entry)**						
Number of emergency visits (mean ± SD[‡])	5.23 ± 4.86	4.74 ± 5.85	0.05	3.28 ± 2.79	3.32 ± 3.29	0.01
Number of all-cause hospital admission (mean ±SD[‡])	2.46 ± 1.96	2.20 ± 2.05	0.13	2.34 ± 1.90	2.49 ± 2.11	0.05

*: BID: Twice daily;[‡]: SD: Standard deviation; [¥]: Acetylsalicylic acid: ASA; [€]: IPTW: Inverse-probability treatment-weighting: Nonsteroidal anti-inflammatory drugs: NSAIDs.

Supplemental Table S9. Rate of clinical events of warfarin vs rivaroxaban 20 mg during 1-year period of follow-up using inverse-probability-of-treatment-weighting.

	Intention-to-treat		Under treatment	
	Warfarin (n=1,253)	Rivaroxaban 20 mg† (n=403)	Warfarin (n= 1,253)	Rivaroxaban 20 mg† (n=403)
**Intracranial bleeding**				
Events	5	1	5	0
Event rate per 100 person-years*	0.5	0.2	0.7	0.0
HR (95% CI)	0.48 (0.04 – 5.13), p-value: 0.54		-	
**Hemorrhagic stroke**				
Events	2	0	2	0
Event rate per 100 person-years*	0.2	0.0	0.3	0.0
HR (95% CI)	-		-	
**Gastrointestinal bleeding**				
Events	14	0	11	0
Event rate per 100 person-years*	1.4	0.0	1.4	0.0
HR (95% CI)	-		-	
**Other bleeding**				
Events	14	8	10	8
Event rate per 100 person-years*	1.3	2.5	1.2	3.2
HR (95% CI)	1.94 (0.82 – 4.58), p-value: 0.13		2.60 (1.03 – 6.59), p-value: 0.04	
**Safety composite**				
Events	32	9	26	8
Event rate per 100 person-years*	3.0	2.7	3.3	3.2
HR (95% CI)	0.91 (0.44 – 1.91), p-value: 0.81		0.98 (0.45 – 2.13), p-value: 0.95	
**Stroke**				
Events	10	3	9	3
Event rate per 100 person-years*	1.0	1.0	1.2	1.0
HR (95% CI)	1.06 (0.31 – 3.60), p-value: 0.92		0.83 (0.21 – 3.26), p-value: 0.79	
**Systemic embolism**				
Events	2	5	2	0
Event rate per 100 person-years*	0.2	1.4	0.2	0.0
HR (95% CI)	8.20 (1.47 – 45.69), p-value: 0.02		-	
**Acute myocardial** infarction				
Events	20	14	17	11
Event rate per 100 person-years*	1.9	4.2	2.2	4.1
HR (95% CI)	2.19 (1.11 – 4.30), p-value: 0.02		1.89 (0.88 – 4.04), p-value: 0.10	
**Death**				
Events	69	20	22	4
Event rate per 100 person-years*	6.4	5.9	2.8	1.5
HR (95% CI)	0.92 (0.56 – 1.51), p-value: 0.73		0.53 (0.18 – 1.55), p-value: 0.25	
**Irreversible events**				
Events	95	39	48	17
Event rate per 100 person-years*	9.0	11.5	6.2	6.6
HR (95% CI)	1.27 (0.88 – 1.85), p-value: 0.21		1.07 (0.62 – 1.86), p-value: 0.80	
**Composite effectiveness**				
Events	94	43	47	17
Event rate per 100 person-years*	8.9	12.6	6.1	6.6
HR (95% CI)	1.42 (0.99 – 2.04), p-value: 0.06		1.10 (0.63 – 1.90), p-value: 0.74	

†: DIE *Crude analysis; HR: hazard ratio; CI: confidence interval.

Supplemental Table S10. Rate of clinical events of warfarin vs apixaban 5 mg during 1-year period of follow-up using inverse-probability-of-treatment-weighting.

	**Intention-to-treat**		**Under treatment**	
	Warfarin (n=1,253)	Apixaban 5 mg* (n=539)	Warfarin (n= 1,253)	Apixaban 5 mg* (n=539)
**Intracranial bleeding**				
Events	5	0	5	0
Event rate per 100 person-years*	0.5	0.0	0.7	0.0
HR (95% CI)	-		-	
**Hemorrhagic stroke**				
Events	2	0	2	0
Event rate per 100 person-years*	0.2	0.0	0.3	0.0
HR (95% CI)	-		-	
**Gastrointestinal bleeding**				
Events	15	2	11	1
Event rate per 100 person-years*	1.4	0.4	1.4	0.3
HR (95% CI)	0.31 (0.07 – 1.35), p-value: 0.12		0.23 (0.03 – 1.60), p-value: 0.14	
**Other bleeding**				
Events	13	4	9	4
Event rate per 100 person-years*	1.2	0.8	1.2	1.0
HR (95% CI)	0.61 (0.19 – 1.99), p-value: 0.42		0.84 (0.25 – 2.85), p-value: 0.77	
**Safety composite**				
Events	32	6	25	5
Event rate per 100 person-years*	3.0	1.2	3.3	1.3
HR (95% CI)	0.40 (0.16 – 0.98), p-value: 0.05		0.40 (0.15 – 1.07), p-value: 0.07	
**Stroke**				
Events	11	3	10	3
Event rate per 100 person-years*	1.0	0.7	1.3	0.9
HR (95% CI)	0.75 (0.22 – 2.53), p-value: 0.64		0.78 (0.23 – 2.67), p-value: 0.69	
**Systemic embolism**				
Events	2	1	2	1
Event rate per 100 person-years*	0.4	0.2	0.5	0.2
HR (95% CI)	1.09 (0.08 – 14.15), p-value: 0.95		1.03 (0.08 – 13.43), p-value: 0.98	
**Acute myocardial** infarction				
Events	20	14	17	11
Event rate per 100 person-years*	1.9	3.0	2.2	3.1
HR (95% CI)	1.54 (0.78 – 3.05), p-value: 0.21		1.43 (0.67 – 3.03), p-value: 0.35	
**Death**				
Events	70	24	22	6
Event rate per 100 person-years*	6.7	5.1	2.8	1.7
HR (95% CI)	0.77 (0.48 – 1.22), p-value: 0.26		0.62 (0.26 – 1.52), p-value: 0.30	
**Irreversible events**				
Events	97	40	49	21
Event rate per 100 person-years*	9.3	8.5	6.3	5.8
HR (95% CI)	0.93 (0.64 – 1.34), p-value: 0.68		0.94 (0.56 – 1.57), p-value: 0.81	
**Composite effectiveness**				
Events	96	41	47	22
Event rate per 100 person-years*	9.1	8.7	6.1	6.0
HR (95% CI)	0.96 (0.67 – 1.39), p-value: 0.82		1.00 (0.60 – 1.66), p-value: 0.99	

†: DIE *Crude analysis; HR: hazard ratio; CI: confidence interval.
